# The locus coeruleus contributes to the anorectic, nausea, and autonomic physiological effects of glucagon-like peptide-1

**DOI:** 10.1126/sciadv.adh0980

**Published:** 2023-09-20

**Authors:** Samantha M. Fortin, Jack C. Chen, Marisa C. Petticord, Forrest J. Ragozzino, James H. Peters, Matthew R. Hayes

**Affiliations:** ^1^ Department of Psychiatry, University of Pennsylvania, Philadelphia, PA 19104, USA.; ^2^ Department of Integrative Physiology and Neuroscience, Washington State University, Pullman, WA 99164, USA.

## Abstract

Increasing the therapeutic potential and reducing the side effects of U.S. Food and Drug Administration–approved glucagon-like peptide-1 receptor (GLP-1R) agonists used to treat obesity require complete characterization of the central mechanisms that mediate both the food intake-suppressive and illness-like effects of GLP-1R signaling. Our studies, in the rat, demonstrate that GLP-1Rs in the locus coeruleus (LC) are pharmacologically and physiologically relevant for food intake control. Furthermore, agonism of LC GLP-1Rs induces illness-like behaviors, and antagonism of LC GLP-1Rs can attenuate GLP-1R–mediated nausea. Electrophysiological and behavioral pharmacology data support a role for LC GLP-1Rs expressed on presynaptic glutamatergic terminals in the control of feeding and malaise. Collectively, our work establishes the LC as a site of action for GLP-1 signaling and extends our understanding of the GLP-1 signaling mechanism necessary for the development of improved obesity pharmacotherapies.

## Teaser

The locus coeruleus contributes to the therapeutic and side effects of glucagon-like peptide-1 (GLP-1) signaling.

## INTRODUCTION

Obesity currently affects 42% of adults and 19% of children in the United States (*1*), creating an enormous health and economic burden (*2*, *3*). Effective and well-tolerated anti-obesity drugs are desperately needed to combat the obesity epidemic, as behavioral strategies offer limited success in the overwhelming majority of individuals with obesity (*4*–*6*). The utilization of obesity pharmacotherapies targeting glucagon-like peptide-1 receptors (GLP-1R) have had clinical success (*7*, *8*) and are U.S. Food and Drug Administration (FDA)–approved for diabetes and obesity treatment (e.g., liraglutide and semaglutide). However, despite their hypophagic effects in human and animal models (*8*–*10*), all clinically approved GLP-1R agonists are burdened with compliance barriers attributed to side effects including nausea, vomiting, and malaise (*11*–*14*). Increasing the therapeutic potential of GLP-1R agonists therefore involves a twofold approach: maximizing the food intake and body weight–suppressive effects of GLP-1R ligands while minimizing nausea and emesis. To achieve these goals, basic research is needed to characterize the mechanisms and neural substrates that underly the full extent of the physiological and behavioral responses to GLP-1 signaling.

The control of food intake and the induction of nausea and vomiting involve complex interactions between distinct and overlapping neural substrates and anatomical nodes in the brain (*15*–*18*). Both the hypophagic and nausea/emesis effects of GLP-1R agonists require activation of central GLP-1Rs (*14*, *19*–*23*). A multitude of studies, including those of our laboratory (*23*–*26*), have shown the contribution of selective GLP-1R populations across neuroanatomically distributed nuclei of the brain in mediating various effects of systemic GLP-1R agonists (*27*–*31*). Although hindbrain preproglucagon (PPG) neurons that synthesize and release GLP-1 project directly to the locus coeruleus (LC) (*32*), the role of the LC in mediating GLP-1’s effects on food intake control has not been examined.

The LC is a highly conserved brainstem nucleus that comprises a primary source of norepinephrine (NE) in the brain (*33*). While there is no unified and comprehensive role for the LC NE system in physiology and behavior, evidence suggests that LC NE neurons contribute to aspects of arousal and autonomic processing (*34*). Recently, the role of the LC in modulating food intake has also been demonstrated (*35*, *36*); however, the mechanisms and neural substrates within the LC remain largely uninvestigated. Here, we demonstrate a role for LC GLP-1R signaling in the control of food intake, autonomic physiology, and visceral malaise. Our data suggest that presynaptic glutamate terminals in the LC not only are a site of action associated with the anorectic, illness-like, and autonomic effects of endogenous GLP-1R signaling but also mediate, in part, the food intake suppressive and illness-like effects of semaglutide and exendin-4 (Ex-4), FDA-approved GLP-1R agonists. Our work aids in dissociating the neural substrates that mediate the anorectic effects from those that contribute to the feeling of illness following either endogenous GLP-1R signaling or delivery of GLP-1R agonists for obesity and diabetes treatment.

## RESULTS

### LC GLP-1R activation controls food intake

**Fig. 1.**
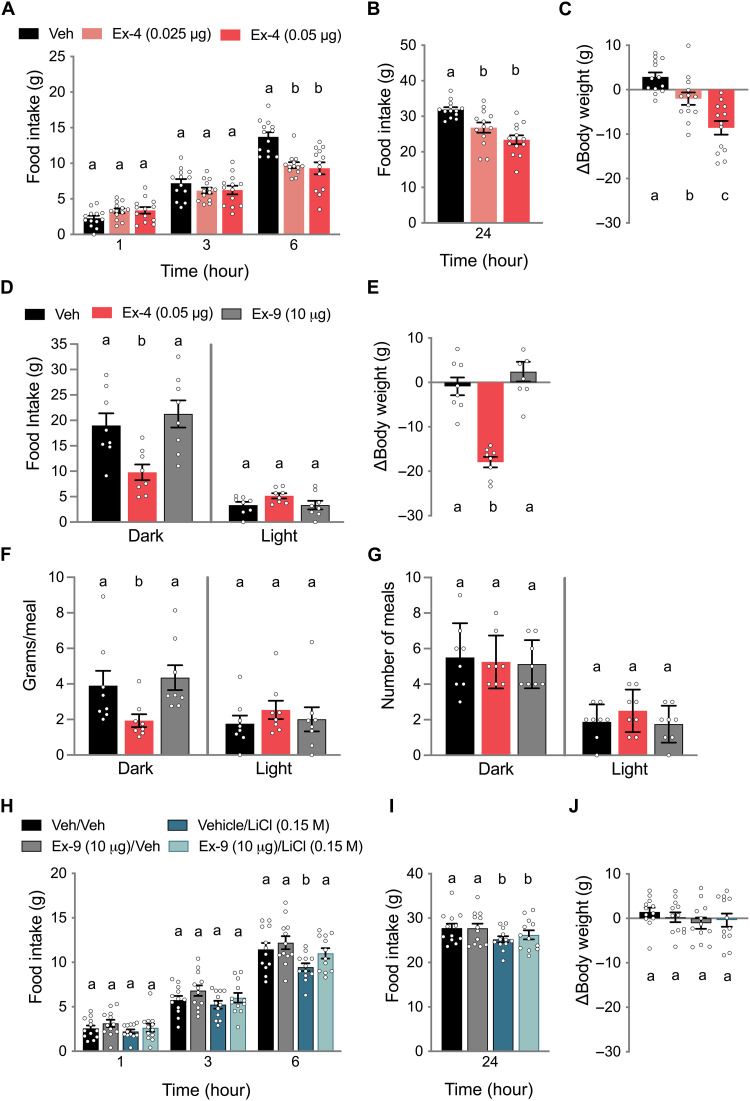
LC GLP-1Rs mediate food intake and body weight control. (**A**) Direct administration of Ex-4 to the LC reduced chow intake at 6 hours after administration (*n* = 13). (**B**) LC Ex-4 suppressed food intake at 24 hours after administration (*n* = 13). (**C**) LC Ex-4 had a dose-dependent effect on body weight suppression measured 24 hours after administration (*n* = 13). (**D**) In a separate cohort of rats (*n* = 8), 0.05 μg of Ex-4 suppressed only dark cycle intake, while Ex-9 (10 μg) had no effect on dark or light cycle intake. (**E**) Body weight measured over 24 hours was reduced by LC Ex-4 and not affected by LC Ex-9 (*n* = 8). (**F**) Changes in food intake during the dark cycle by LC Ex-4 were mediated by a reduction in meal size (*n* = 8). (**G**) The number of meals consumed during either the dark or light cycle was not affected by LC Ex-4 or LC Ex-9 (*n* = 8). (**H**) Food intake suppression by LiCl (0.15 M; 20 ml/kg) at 6 hours was attenuated by LC Ex-9 (*n* = 12). (**I**) Attenuation of LiCl-induced food intake suppression at 24 hours was not observed at 24 hours (*n* = 12). (**J**) No effects on 24-hour body weight change were observed following LiCl or Ex-9 treatment combinations (*n* = 12). All data are expressed as means ± SEM. Data in (A) to (G) were analyzed with a repeated-measures one-way analysis of variance (ANOVA) at each time point followed by Newman Keuls post hoc tests. Data in (H) to (J) were analyzed with repeated-measures two-way ANOVA followed by Newman-Keuls post hoc tests. Means with different letters are significantly different from each other (*P* < 0.05). Veh, vehicle.

To begin to examine the role of LC GLP-1R signaling in food intake control, rats implanted with an indwelling cannula aimed at the LC were injected with the GLP-1R agonist Ex-4. At doses subthreshold for feeding effects when delivered to the ventricle (*20*, *30*, *37*), LC Ex-4 reduced chow intake at 6 hours after administration relative to vehicle-injected animals (Fig. 1A). A dose-response reduction in feeding behavior (Fig. 1B) and body weight loss (Fig. 1C) was observed at 24 hours.

In a separate cohort of rats, a continuous food intake monitoring BioDAQ system was used to establish changes in meal patterns with LC Ex-4 administration. Rats were also tested with the GLP-1R antagonist, exendin (9-39) (Ex-9), to investigate an endogenous contribution of LC GLP-1R signaling to feeding behavior. Only during the 12-hour dark cycle, in which rats naturally feed, did Ex-4 suppress overall food intake relative to vehicle treatment (Fig. 1D), recapitulating the effect observed in Fig. 1, A and B. Body weight over the 24-hour post-administration period was decreased by Ex-4 but unaffected by Ex-9 treatment (Fig. 1E). The effect of Ex-4 on food intake was driven by a reduction in dark cycle meal size (Fig. 1F) but not meal number (Fig. 1G).

While the dose of intra-LC Ex-9 used (10 μg/100 nl) to block endogenous LC GLP-1R activity had no effect on food intake or body weight (Fig. 1, D to F), LC administration of Ex-9 at the same dose attenuated the food intake reduction caused by the illness-inducing agent lithium chloride (LiCl), a substance demonstrated to activate hindbrain PPG neurons (*38*, *39*), at 6 hours (Fig. 1H). While cumulative food intake following LiCl was still suppressed at 24 hours, attenuation of food intake suppression by LC Ex-9 was absent at this later time point (Fig. 1I). There were no effects of peripheral LiCl or LC Ex-9 on 24-hour body weight (Fig. 1J).

### LC GLP-1R activation drives select autonomic responses

**Fig. 2.**
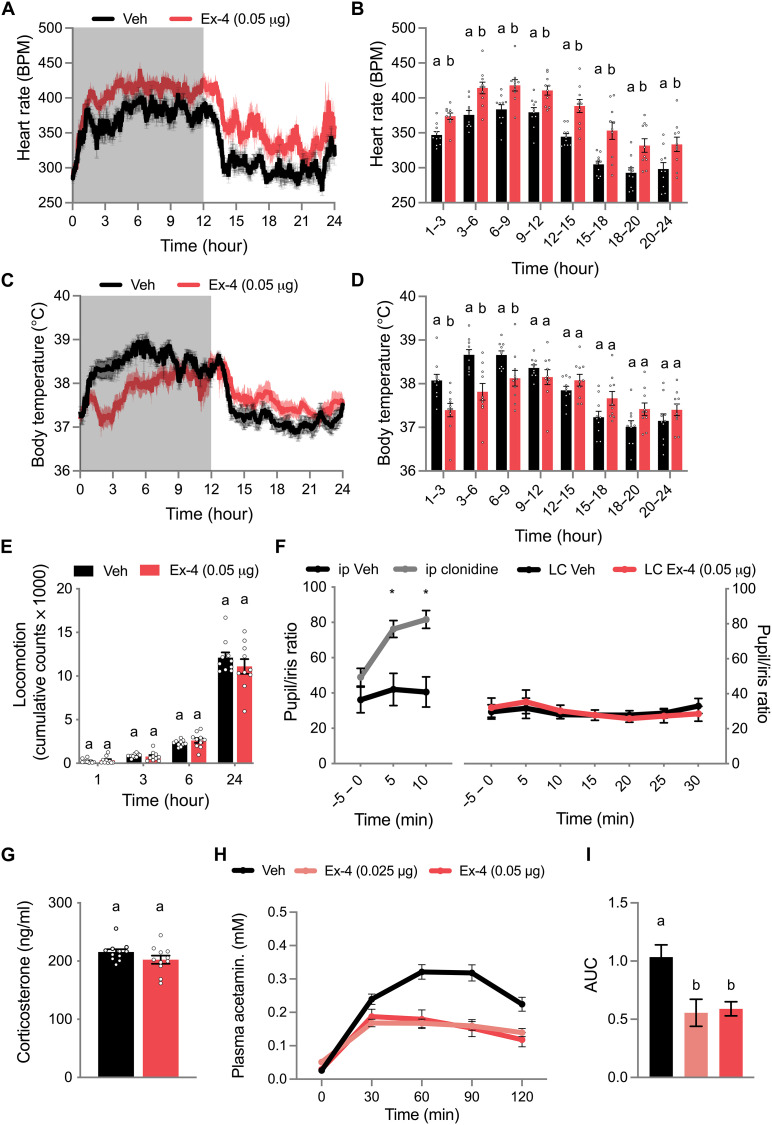
LC GLP-1Rs mediate tachycardia, hypothermia, and gastric emptying rate. (**A**) LC Ex-4, injected at the onset of the 12-hour dark cycle (gray), induced tachycardia relative to vehicle-treated rats (*n* = 10). (**B**) When quantified in 3-hour bins, LC Ex-4 induced tachycardia at all measured time points over 24 hours (*n* = 10). (**C**) LC Ex-4, injected at the onset of the 12-hour dark cycle (gray), induced hypothermia relative to vehicle-treated rats (*n* = 10). (**D**) When quantified in 3-hour bins, LC Ex-4 induced hypothermia during the first 9 hours after administration. (**E**) LC Ex-4 did not change cumulative locomotor counts over 24 hours (*n* = 10). (**F**) Intraperitoneal (ip) injection of clonidine (0.1 mg/kg) significantly increased pupil dilation relative to vehicle-injected rats (left; *n* = 5). However, LC Ex-4 (0.05 μg) did not affect pupil dilation (right) (*n* = 7). (**G**) LC Ex-4 did not affect plasma corticosterone release at 90 min after administration (*n* = 11). (**H**) The gastric emptying rate of gavaged acetaminophen in Ensure (40 mg/6 ml) was reduced by LC Ex-4 relative to vehicle-treated rats (*n* = 14). (**I**) When quantified as an area under the curve (AUC), plasma acetaminophen concentrations were reduced by both 0.025 and 0.05 μg of Ex-4 (*n* = 14). All data were expressed as means ± SEM. Binned data in (B) and (D) and data in (E) to (G) were analyzed with Student’s *t* test at each time point. Data in (I) were analyzed with a repeated-measures one-way ANOVA followed by Newman-Keuls post hoc tests. Means with different letters are significantly different from each other (*P* < 0.05) and *P < 0.05. BPM, beats per minute.

As the LC has an established role in coordinating autonomic responses (*34*), we next examined the ability of LC Ex-4 to affect sympathetic and parasympathetic outflow. LC Ex-4 caused a marked increase in heart rate (HR) relative to vehicle injection (Fig. 2A). When quantified in three-hour increments over 24 hours, intra-LC Ex-4–induced tachycardia was significant at all measured time intervals relative to HR following vehicle injections (Fig. 2B). Accompanying an increase in HR was a decrease in body temperature by intra-LC Ex-4 (Fig. 2C) that was significant for the first 9 hours after injection (Fig. 2D). Overall, locomotor activity of the rats was not affected by LC Ex-4 (Fig. 2E). While clonidine, administered at a dose known to alter HR and body temperature (*40*), induced activation of LC NE neurons increased pupil dilation, as previously reported (*41*), LC Ex-4 had no effect on pupil to iris ratio (Fig. 2F). Plasma corticosterone at 90 min after administration was also not affected by LC Ex-4 (Fig. 2G). To measure the effects of LC Ex-4 on the regulation of gastric emptying, we conducted an enzyme-linked immunosorbent assay (ELISA) to detect acetaminophen in the plasma following oral gavage, given that acetaminophen is only absorbed once it passes into the duodenum (*42*, *43*). Both LC-administered doses of Ex-4 delayed gastric emptying as shown by a decrease in the rate of acetaminophen detected in the blood over the course of 2 hours (Fig. 2H), resulting in a significant reduction in area under the curve for both doses relative to vehicle-injected animals (Fig. 2I). These data identify a central nervous system (CNS) nucleus mediating the seldom reported autonomic responses of acute GLP-1R agonism and suggest a neural substrate (i.e., GLP-1) that is capable of engaging LC neural activity to affect core body temperature, gastric emptying, and HR.

### LC GLP-1R activation produces illness-like behavior

**Fig. 3.**
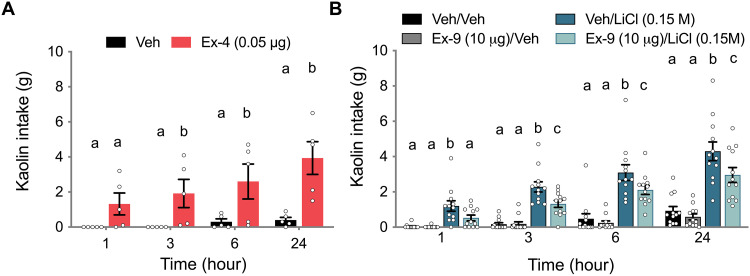
LC GLP-1Rs mediate kaolin consumption. (**A**) Direct administration of Ex-4 to the LC induced an increase in kaolin intake (*n* = 5) relative to vehicle-treated rats (*n* = 5) at 3, 6, and 24 hours after administration. (**B**) Kaolin intake induced by LiCl at 1, 3, 6, and 24 hours was attenuated by bilateral LC Ex-9 (10 μg/100 nl) (*n* = 12). All data were expressed as means ± SEM. Data in (A) were analyzed with a repeated-measures one-way ANOVA at each time point followed by Newman-Keuls post hoc tests. Data in (B) were analyzed with repeated-measures two-way ANOVA followed by Newman-Keuls post hoc tests. Means with different letters are significantly different from each other (*P* < 0.05).

Given the reduction in gastric emptying by LC Ex-4, we next examined whether LC Ex-4 induced pica, the consumption of nonnutritive kaolin clay, a well-documented behavior indicative of nausea/malaise that has been shown to accompany gastric stasis (*44*–*46*). LC Ex-4 induced significant increases in kaolin consumption, compared to vehicle-injected rats, at 3, 6, and 24 hours (Fig. 3A). Food intake suppression and pica induced by LiCl, a substance known to cause illness in part through engagement of the central GLP-1 system (*38*, *39*), were attenuated by LC GLP-1R antagonism (Ex-9) pretreatment (Fig. 3B). These data highlight the LC as a CNS nucleus that mediates GLP-1's contribution to LiCl-induced visceral malaise.

### LC GLP-1R activation increases glutamate signaling onto LC NE neurons

**Fig. 4.**
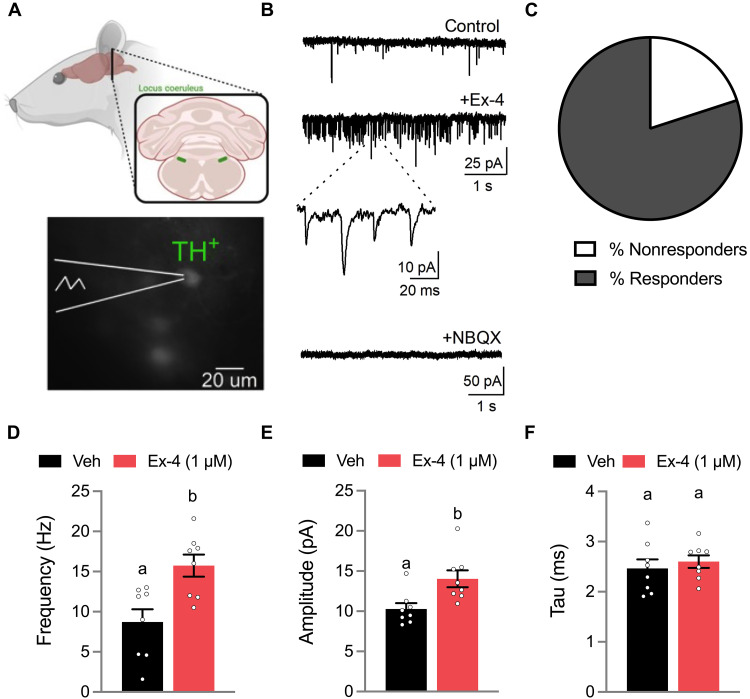
LC GLP-1R activation increases glutamate signaling onto LC NE neurons. (**A**) Voltage-clamp electrophysiology recordings of TH^+^ enhanced green fluorescent protein–labeled LC NE neurons. (**B**) Representative traces showing bath applied Ex-4 (1 μM) increased the frequency of spontaneous excitatory postsynaptic currents (EPSCs). Bath application of the AMPA-type glutamate receptor antagonist 2,3-dihydroxy-6-nitro-7-sulfamoylbenzo[f]quinoxaline (NBQX) eliminated the phasic waveforms. (**C**) The majority of recorded neurons (*n* = 8 of 10, 80%) responded to bath applied Ex-4. (**D**) Ex-4 significantly increased the frequency of spontaneous EPSCs. (**E**) Ex-4 also increased the amplitude of EPSCs in responsive neurons (*n* = 8). (**F**) The decay kinetics (tau) were not affected by Ex-4 application relative to control (*n* = 8). All data were expressed as means (D and F) or median (E) ± SEM. All data were analyzed with paired Student’s *t* tests (D and F) or Wilcoxon signed-rank test (E). Means with different letters are significantly different from each other (*P* < 0.05).

The autonomic and feeding effects of the LC are likely to be mediated by NE neurons, a predominant cell type of the LC. We performed patch clamp electrophysiology of LC NE neurons (tyrosine hydroxylase– expressing, TH^+^), which were visualized using a viral labeling approach, to examine whether LC NE neurons are responsive to Ex-4 (Fig. 4A). Bath application of Ex-4 increased excitatory glutamatergic synaptic activity on to LC NE neurons (Fig. 4B) in 80% of recorded neurons (Fig. 4C). Bath application of the AMPA-type glutamate receptor antagonist 2,3-dihydroxy-6-nitro-7-sulfamoylbenzo[f]quinoxaline (NBQX) eliminated Ex-4–evoked excitatory postsynaptic currents (EPSCs) (Fig. 4B). In these responsive neurons, Ex-4 increased the frequency (Fig. 4D) and amplitude (Fig. 4E) of spontaneous EPSCs, while the decay kinetics (tau) were not altered by Ex-4 (Fig. 4F). In addition to changes in synaptic glutamate release, Ex-4 also produced a small and reversible change in membrane holding current (base: −83 ± 15 pA versus Ex-4: −115 ± 15 pA, paired Student’s *t* test, *P* = 0.01). In a small sampling of non-NE LC neurons (*n* = 3 neurons from 3 rats), Ex-4 showed no effect on EPSC frequency (control: 2.43 ± 0.87 Hz versus Ex-4: 2.83 ± 0.62 Hz, Wilcoxon signed-rank test, *P* = 0.25).

### LC GLP-1R activation contributes to food intake control via enhanced glutamate signaling

**Fig. 5.**
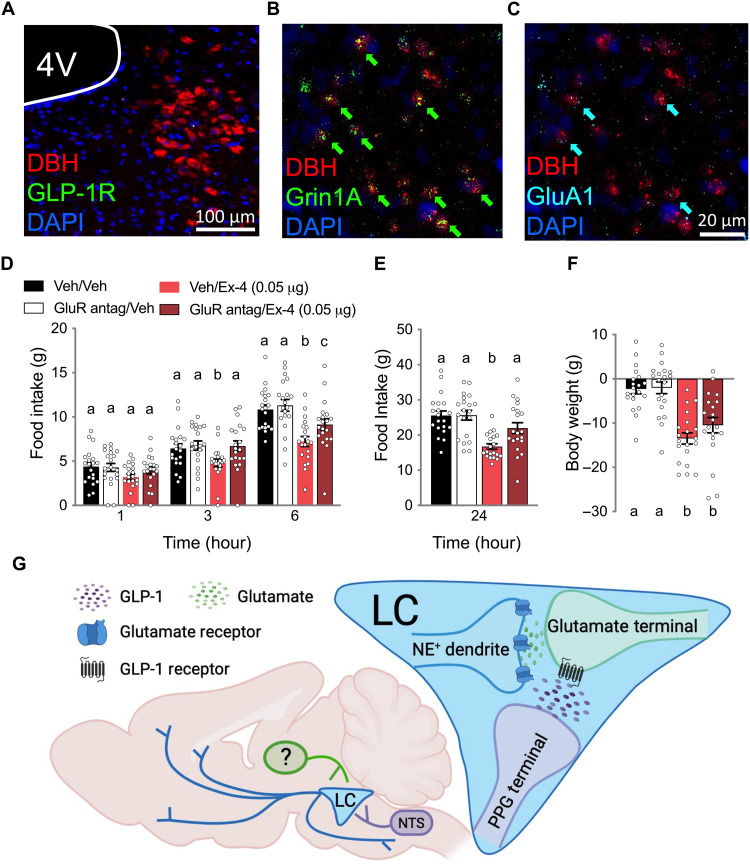
LC GLP-1R activation contributes to food intake control via enhanced glutamate signaling. (**A**) RNAscope analysis did not reveal *Glp1r* mRNA expression in LC NE neuron (DBH) soma. (**B**) LC NE neurons express *N*-methyl-d-aspartate (NMDA) receptors (*Grin1A*). (**C**) LC NE neurons express AMPA receptors (*GluA1*). (**D**) Pretreatment with an NMDA/AMPA antagonist cocktail [GluR antag; MK-801, 0.05 μg/CNQX (6-cyano-7-nitroquinoxaline-2,3-dione), 0.3 μg] attenuated the food intake-suppressive effects of LC Ex-4 (0.05 μg) at 3 and 6 hours after administration (*n* = 20). (**E**) MK-801/CNQX attenuated the food intake suppressive effects of LC Ex-4 at 24 hours (*n* = 20). (**F**) Twenty-four-hour body weight reduction by LC Ex-4 was not significantly affected by MK-801/CNQX pretreatment (*n* = 20). (**G**) Proposed mechanism by which LC GLP-1R activation increases LC NE activity, suppresses food intake, and causes illness-like and autonomic effects. All data were expressed as means ± SEM. Data in (D) to (F) were analyzed with a repeated-measures two-way ANOVA at each time point followed by Newman-Keuls post hoc tests. Means with different letters are significantly different from each other (*P* < 0.05). DAPI, 4′,6-diamidino-2-phenylindole.

In situ hybridization revealed the absence of *Glpr1* mRNA expression in LC NE neuron (dopamine beta-hydroxylase–expressing, DBH^+^) soma (Fig. 5A). Our data demonstrating robust behavioral (Figs. 1 to 3) and electrophysiological (Fig. 4) effects of LC Ex-4, together with established projections from the nucleus tractus solitarius (NTS) PPG neurons to the LC (*32*), led us to consider a presynaptic GLP-1R–mediated mechanism. Consistent with AMPA-mediated increases in LC NE activity by Ex-4 (Fig. 4), in situ hybridization analysis shows that LC NE neurons express both *N*-methyl-d-aspartate (NMDA) (*Grin1A*; Fig. 5B) and AMPA receptor mRNA (*GluA1*; Fig. 5C).

We next tested the contribution of LC glutamate receptors to LC Ex-4–induced anorexia. Blocking LC glutamate signaling with microinjection of an NMDA/AMPA receptor antagonist cocktail [MK-801/CNQX (6-cyano-7-nitroquinoxaline-2,3-dione)] attenuated the food intake suppressive effects of intra-LC delivery of Ex-4 at 3, 6, and 24 hours (Fig. 5, D and E). Twenty-four-hour body weight loss by Ex-4 was not significantly attenuated by LC glutamate receptor antagonism (Fig. 5F). Electrophysiology, in situ hybridization, and behavioral pharmacology data collectively support a mechanism by which GLP-1Rs expressed on presynaptic glutamatergic terminals in the LC contribute to LC NE activity and the behavioral and physiological effects of LC GLP-1R agonism (Fig. 5G).

### Peripheral delivery of semaglutide activates LC NE neurons and LC GLP-1Rs are required for the full food intake and body weight suppressive effects, as well as the illness-like behaviors, of semaglutide

**Fig. 6.**
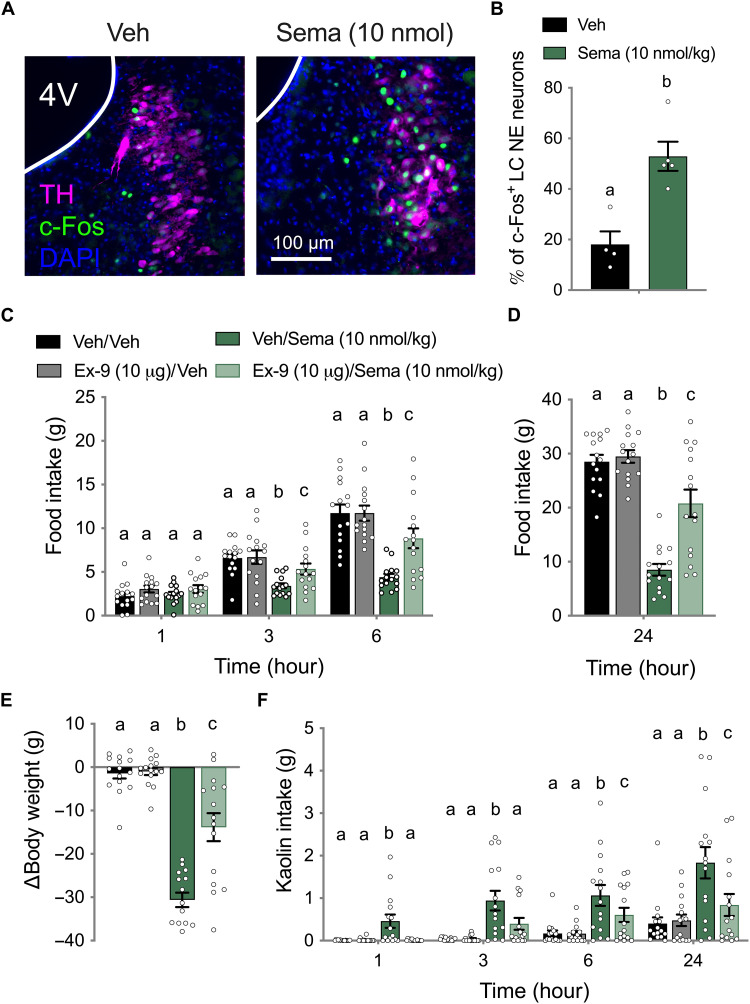
LC GLP-1Rs contribute to the therapeutic and illness-like effects of semaglutide. (**A**) Representative images of increased neural activity (c-Fos; green) of LC NE neurons (TH^+^) by peripheral administration of semaglutide (Sema; 10 nmol/kg; ip) compared to vehicle. (**B**) Quantification revealed a significant increase in the percentage of c-Fos^+^ LC NE neurons in ip semaglutide (*n* = 5) relative to vehicle (*n* = 4) treated rats. (**C**) Food intake suppression by ip semaglutide (10 nmol/kg) at 3 and 6 hours was attenuated by pretreatment with LC Ex-9 (10 μg) (*n* = 15). (**D**) Food intake suppression by ip semaglutide (10 nmol/kg) at 24 hours was attenuated by pretreatment with LC Ex-9 (10 μg) (*n* = 15). (**E**) Similarly, LC Ex-9 (10 μg) attenuated body weight suppression by semaglutide (10 nmol/kg) measured 24 hours after administration (*n* = 15). (**F**) In the same cohort of rats, LC Ex-9 (10 μg) attenuated kaolin intake induced by ip semaglutide (10 nmol/kg) at 1, 3, 6, and 24 hours (*n* = 15). All data were expressed as means ± SEM. Data in (B) were analyzed with a Student’s *t* test. Data in (C) to (F) were analyzed with a repeated-measures two-way ANOVA at each time point followed by Newman-Keuls post hoc tests. Means with different letters are significantly different from each other (*P* < 0.05).

We next measured c-Fos activity in the brains of rats treated with either peripheral administration of a food intake–suppressive dose of semaglutide (10 nmol/kg) (*47*) or vehicle (Fig. 6A). Quantification revealed a significant increase in c-Fos activation of LC NE neurons by semaglutide compared to vehicle treatment (Fig. 6B).

To directly test the contribution of LC GLP-1Rs to the behavioral effects of semaglutide, we examined the effect of LC GLP-1R antagonism on semaglutide-induced food intake and body weight suppression, as well as kaolin intake. Pretreatment with LC Ex-9 attenuated food intake suppression by semaglutide at 3, 6, and 24 hours (Fig. 6, C and D). Semaglutide-induced body weight loss at 24 hours was also attenuated by LC Ex-9 (Fig. 6E). Semaglutide-induced kaolin intake at 1, 3, 6, and 24 hours was attenuated by LC Ex-9 pretreatment (Fig. 6F).

## DISCUSSION

Central GLP-1Rs distributed across the brain are predicted to mediate the anorectic and illness-like effects that accompany the therapeutic benefits of GLP-1–based analogs used for the treatment of diabetes and obesity. However, the neuronal populations mediating the intake versus malaise effects of GLP-1R signaling remain incompletely described (*48*). Thus, we must fully characterize which GLP-1 sites of action mediate nausea/emesis to best develop obesity pharmacotherapies that maintain their therapeutic potential with minimal side effects. To this end, we examined the endogenous and exogenous contributions of GLP-1Rs in the LC to mediate the food intake suppressive and illness-like behaviors characteristic of GLP-1R signaling. Our findings demonstrate that LC GLP-1Rs expressed on presynaptic glutamatergic terminals contribute to the anorectic, autonomic, and illness-like effects driving the net suppressive effects on energy balance by GLP-1R signaling.

We first examined the hypothesis that activation of GLP-1Rs in the LC would suppress food intake and body weight. While there is little physiological evidence for LC GLP-1Rs being involved in food intake control, it is known that hindbrain PPG neurons project directly to the LC (*32*), and recent research has indicated that the LC contributes to aspects of feeding behavior (*35*, *36*). Our initial dose response studies with LC microinjections of Ex-4 revealed that activation of LC GLP-1Rs suppresses food intake at 6 and 24 hours. Food intake suppression by LC Ex-4 resulted in a significant body weight loss measured at 24 hours after administration. To replicate and extend our findings, we similarly injected a separate cohort of rats housed in a BioDAQ system for continuous monitoring of food intake. Meal pattern analysis revealed that the decrease in food intake during the dark cycle, in which rats are awake and consume most of their calories, by LC Ex-4 is mediated by a reduction in meal size and not meal number. These findings are consistent with GLP-1R activation in other areas of the brain (*20*, *24*, *26*, *29*), which have collectively contributed to our appreciation of the central GLP-1 system as one that is responsive to not only within-meal satiation signals but also stressors (e.g., visceral malaise, restraint, LiCl, and LPS) (*49*–*52*). In our within-subjects design, we also administered the GLP-1 antagonist Ex-9 to probe the endogenous relevance of LC GLP-1Rs. We found that LC Ex-9 (*25*, *31*) application had no effect on food intake, meal patterns, or body weight change. As hindbrain GLP-1–producing neurons are robustly activated by a large meal or visceral stressor (*51*–*53*), we next examined the endogenous contribution of LC GLP-1Rs when NTS PPG neurons were engaged by the visceral toxin LiCl. As expected, LiCl caused a reduction in food intake (*54*). Food intake reduction by LiCl was attenuated by LC pretreatment with Ex-9. Future work antagonizing LC GLP-1Rs following chemogenetic activation of PPG neurons or scenarios known to initiate central GLP-1 release (e.g., fasting-induced refeeding, gastric distention, and stress) will be needed to further characterize the endogenous relevance of LC GLP-1Rs. However, these data indicate that LC GLP-1Rs contribute to the satiation effects of exogenous GLP-1R ligands and are suggestive of mediating food intake reduction following central GLP-1 release.

The LC has an established role in mediating autonomic nervous system output through NE release throughout the brain (*34*). In addition to wakefulness, the LC NE system is broadly involved with the regulation of cardiovascular, gastrointestinal, and urogenital systems (*34*). We hypothesized that food intake reduction by LC GLP-1R signaling was antecedent to changes in sympathetic and parasympathetic outflow. In rats implanted with telemetric transponders, we observed an increase in HR and a decrease in body temperature following LC GLP-1R stimulation with no significant change in locomotion. These seemingly paradoxical responses have been reported previously with systemic or ICV delivery of Ex-4 (*55*–*57*). Chemogenetic activation of PPG neurons has shown that central release of GLP-1 increases resting HR (*57*), and chronic decerebrate preparations in rats suggest that hindbrain-restricted nuclei mediate the sympathetic effects of GLP-1 signaling (*19*). Here, we highlight a specific nucleus site of action that is sufficient to mediate the tachycardic and hypothermic effects of central GLP-1R signaling.

In addition to tachycardia and hypothermia, both central GLP-1 signaling (*58*–*60*) and the LC (*61*, *62*) have been implicated in the regulation of gastrointestinal motility. Using an established paradigm for measuring gastric emptying in the rat (*42*), we observed that LC Ex-4 delays the transport of acetaminophen from the stomach to the small intestine. Our data suggest that GLP-1R action in the LC contributes to the delay in gastric emptying by GLP-1R agonism observed in previous studies. As reductions in gastric emptying contribute to visceral malaise (*63*), a symptom that has been shown to accompany GLP-1R signaling (*14*), we next investigated whether reductions in food intake by LC Ex-4 were accompanied by illness-like effects. To study visceral illness in a rodent species that is incapable of vomiting, our laboratory and others measure the intake of aluminum silicate (kaolin, e.g., pica—the ingestion of nonnutritive substances in response to the feeling of visceral malaise) as a proxy for nausea (*46*). We observed that LC Ex-4 stimulates kaolin intake in rats, suggesting that reductions in food intake following LC GLP-1R agonism are, in part, due to feelings of illness. To examine the endogenous contribution of LC GLP-1Rs to visceral malaise, we induced illness with the administration of LiCl, a toxin shown to activate hindbrain PPG neurons (*38*, *39*). LiCl caused kaolin intake in rats that was attenuated by LC pretreatment with Ex-9, suggesting that the LC is among one of a select few nuclei that have been identified to drive illness-like behaviors via the GLP-1 system (*9*, *16*, *64*). The LC has been shown to be both responsive to visceral input including noxious stimuli (*65*–*68*) and stomach distention (*65*, *69*, *70*). Our data suggest that feelings of visceral malaise, potentially through stasis of the gut, contribute to these observed behaviors.

While our data support delayed gastric emptying and nausea contributing to the anorexia observed following LC GLP-1R activation, other factors may also be at play. Future work will examine the contribution of LC GLP-1Rs to LC NE signaling–induced changes in stress, anxiety, and aversion (*71*–*73*) in addition to arousal (*74*), foraging behavior (*75*), and sensory encoding that includes taste perception (*76*, *77*); all of which may contribute to the observed food intake reduction following GLP-1R activation in the LC. Given that hindbrain PPG neurons are activated by stress including gastric distention and visceral malaise (*50*), it is possible that the LC mediates a range of responses to homeostatic challenge. Thus, the working hypothesis here is that hindbrain PPG neurons respond to stress-mediated inputs (*50*, *78*, *79*), promoting the subsequent release of GLP-1 onto GLP-1Rs in the LC. Voltage-clamp electrophysiology experiments of NE LC neurons demonstrated that GLP-1R signaling is capable of amplifying constitutive glutamatergic neurotransmission onto LC NE neurons. While glutamatergic signaling in the LC has been shown to enhance LC NE signaling (*80*) and GLP-1Rs increase the magnitude of glutamatergic signaling in other areas of the brain (*26*), our findings extend this mechanism and demonstrate GLP-1R modulation of LC NE neuron activity.

The undetected expression of GLP-1Rs on LC NE neurons, consistent with data reported in the mouse (*81*), suggests that relevant GLP-1Rs are located presynaptically, where fluorescence in situ hybridization (FISH) detection would fail to visualize GLP-1R mRNA above background fluorescence. In the current working model, stimulation of GLP-1Rs on presynaptic glutamate terminals facilitates glutamate release onto LC NE neurons, as supported by the current behavioral data and observations of increased spontaneous glutamate release frequency with GLP-1R agonism. However, it should be noted that there remains a potential postsynaptic mechanism that may contribute to the observed effects. Although variable, there was a statistically significant increase in an inward holding current in responsive neurons that partially reversed following wash. This current may result from direct GLP-1R signaling in the LC neurons or be secondary to the ambient increase in glutamate acting via metabotropic receptors. In addition to the change in holding current, we also observed an increase in the spontaneous glutamate EPSC waveform amplitude. This change may be due to an increase in postsynaptic AMPA receptor insertion; however, given the fast temporal dynamics of observed changes in EPSC frequency, it is likely that the change in amplitude resulted from the recruitment of glutamate terminals with larger quantal size that were not initially active. Thus, while there are clear presynaptic neurophysiological changes due to GLP-1R activation, additional studies will be required to dissect possible direct postsynaptic effects of GLP-1R signaling in LC neurons.

Additional work will also be required to identify the source of the glutamatergic input into the LC that is modulated by GLP-1R activation. The LC receives input from over 100 nuclei of the brain (*82*) that are organized in a modular fashion and recruited in an activity-dependent manner (*83*). Sources of glutamatergic inputs previously described to excite LC NE neurons include the prefrontal cortex (*84*) and the paragigantocellular nucleus of the ventrolateral medulla (*85*), although other glutamatergic inputs should be considered (*82*). One candidate nuclei, the parabrachial nucleus (PBN), is a site of converging sensory and visceral malaise signals (*86*), sends direct glutamatergic projections to the LC (*87*), and expresses the GLP-1R (*88*). Other potential sources of glutamate include corticotropin-releasing factor neuron projections from the amygdala or orexin neuron projections from the hypothalamus, both of which co-release glutamate and have been shown to play a role in feeding behavior (*89*, *90*).

Revealing which LC NE projection targets are engaged by LC GLP-1 signaling will be another critical extension of our work. However, this task is challenged by the complexity of LC NE connectivity. Outputs of LC neurons are heterogeneous discrete subpopulations of neurons that are believed to affect distinct aspects of physiology and behavior (*91*). In support of this idea, we have shown that LC Ex-4 impacts select measures of autonomic physiology. However, measures of arousal known to be modulated by LC activity, including locomotion and pupil dilation (*73*, *92*), were not altered by LC GLP-1R signaling. Similarly, despite literature describing LC mediation of HPA axis activity (*93*), and the well-documented role of the GLP-1 system in the stress response (*49*, *94*, *95*), LC Ex-4 does not affect plasma corticosterone levels sampled at 90 min. We chose to sample corticosterone at 90 min as studies in the rat have shown that plasma corticosterone levels begin to rise immediately in response to stressors and central GLP-1 administration, with observable differences measured between 30 and 90 min (*28*, *96*–*99*). A complete time course of plasma corticosterone levels following LC Ex-4 is required to conclude that LC GLP-1Rs do not play a role in LC regulation of HPA axis activity; however, the data presented here support the idea that LC GLP-1R signaling selectively activates a subset of LC NE neurons that control particular aspects of autonomic physiology and behavior.

With respect to feeding behavior, the LC has been shown to modulate aspects of feeding through projections to rostral structures including, but not limited to the lateral hypothalamus (*35*), basolateral amygdala (*72*), and PBN (*36*). As our data support delayed gastric emptying and feelings of illness contributing to anorexia, we are focused on projections with relevance to gut motility including LC NE projections to the dorsal motor nucleus of the vagus (DMV), nucleus ambiguous, raphe, and paraventricular nuceleus of the hypothalamus (PVN) (*34*). Rats without a forebrain terminate feeding in response to GLP-1R agonists, suggesting that the structures necessary for GLP-1–induced meal termination are endemic to the hindbrain (*19*). As the DMV receives LC input (*100*), expresses adrenergic receptors that mediate visceral illness (*101*, *102*), and contributes to the control of gastric motility (*61*, *62*), we believe that LC NE stimulation by GLP-1R signaling results in a delay in gastric emptying and nausea through projections to visceral output structures including the DMV.

While LC activation has been shown to increase HR (*103*–*105*), the neuropeptide/neurotransmitter inputs to the LC NE neurons that mediate this effect are unclear. In addition to causing gastrointestinal distress, LC NE projections to the DMV may also contribute to the cardiovascular effects that we observed with LC GLP-1R activation. It has been shown that while DMV neurons reduce HR when stimulated by glutamate, NE inhibits DMV neurons leading to an increase in HR. Other LC NE targets that may contribute to the tachycardic effects of LC Ex-4 include the nucleus ambiguous, rostral ventrolateral medulla (RVLM), and PVN, all of which receive input from the LC and control cardiovascular function (*106*–*109*). Regulation of body temperature by the LC has been suggested to involve transsynaptic input to the intermediolateral nucleus (IML) of the spinal cord via serotonergic projections of the raphe pallidus (*34*); however, these projections in the context of our work require future study. Investigating the NE outputs that are engaged by GLP-1 is important not only for understanding the negative side effects of obesity pharmacotherapies but also for revealing the LC circuits that contribute to anxiety and stress disorders as well as gastrointestinal dysfunctions observed in Alzheimer’s disease (*110*) and opioid withdrawal (*111*), both of which are believed to involve the LC.

Microinjections of Ex-9 in the LC attenuated the effects of semaglutide on feeding behavior, body weight loss, and illness-like behaviors. These data suggest that LC GLP-1Rs may be a relevant site of action for therapeutic and illness-like effects of peripherally delivered semaglutide. However, the mechanism by which peripherally delivered semaglutide accesses the brain, and how these mechanisms differ from those of endogenous GLP-1 signaling, requires further investigation. The LC is neuroanatomically located immediately anterolateral to the floor of the fourth ventricle. The LC has vast pericoerulear dendritic fields capable of exciting LC NE neurons (*112*) that juxtapose the ependymal cell layer of the fourth ventricle (*113*). Tanycytes in the ependymal layer make contact with LC NE neurons and dendrites (*89*, *114*, *115*) and have been proposed as a possible mechanism for the transport of energy balance–relevant signals to the LC (*116*). We hypothesize that LC NE neuron activation observed following peripheral semaglutide is subsequent to semaglutide accessing GLP-1Rs via fourth ventricular tanycytes. Studies of fluorescently labeled semaglutide and liraglutide have concluded that tanycytes accumulate GLP-1 analogs and provide a mechanism for their transport into the brain (*47*, *117*, *118*). Unfortunately, fourth ventricular tanycytes in close proximity to the LC were not specifically investigated for GLP-1 analog uptake (*47*, *118*). Gabery *et al*. (*47*) did not highlight the LC as a major site of fluorescent-semaglutide accumulation; however, we question how the compound would appear within the diffuse pericoerulear glutamate terminals of the LC where we believe presynaptic GLP-1Rs to be expressed. Future studies focused on investigating the penetrance of GLP-1 analogs into the LC, and the brain in general, perhaps by using fluorescent probes capable of super-resolution microscopy (*119*), is warranted to clarify the clinical relevance of semaglutide signaling in the LC.

Our data contribute to a growing body of evidence that highlights the LC as a feeding-relevant nucleus. As the LC is known to express receptors for other neuropeptides relevant to energy balance regulation, future investigations aimed at characterizing the endogenous mechanism by which the LC participates in food intake control are warranted. Here, we characterize a previously unexplored site of action for endogenous and exogenous GLP-1 signaling. We show that LC GLP-1R activation suppresses food intake, engages autonomic responses, and results in illness-like behaviors. This insight is necessary to advance clinical strategies for the treatment of obesity with improved GLP-1 analogs, with the hope of mitigating the nausea pervasive to current existing GLP-1–based pharmacotherapies.

## MATERIALS AND METHODS

### Experimental model and subject details

Male Sprague-Dawley rats (weighing 325 to 350 g at arrival; Charles River Laboratories) were used for all experiments except electrophysiology experiments, in which TH-cre rats (Envigo) were used. Each distinct experiment was conducted in a separate group of rats. Rats were housed individually in hanging wire mesh cages in temperature/humidity-controlled rooms (12-hour light/dark). All rats had ad libitum access to chow (Purina 5001) and water. In kaolin experiments, rats also had ad libitum access to kaolin pellets (Research Diets, K50001) beginning at least 5 days before the start of the experiment. All surgical procedures were performed under intraperitoneal (ip) anesthesia [xylazine (AnaSed, 2.7 mg/kg), ketamine (Butler Schein, 90 mg/kg), acepromazine (Midwest Veterinary Supply, 0.64 mg/kg); 0.1 ml/100 g body weight] and analgesic [Metacam (Midwest Veterinary Supply, 2 mg/kg, sc)]. Surgical manipulations included implantation of a bilateral guide cannula over the LC (−9.84 mm from bregma, +/− 1.2 mm from midline, 5.2 mm ventral from skull) or implantation of a telemetric transponder (HRC 4000 VitalView; STARR Life Sciences) into the body cavity of the rat. HR leads were secured to the chest muscles on either side of the heart with sutures. Following experimentation, all animals that were used for LC microinjections during experimentation were assessed for proper LC cannula placement. Before euthanasia, rats were injected with 100 nl per hemisphere (volume matched to that of experimental injections) of pontine sky-blue ink. Animals were euthanized and brainstems were coronally sliced at 30 μm using a cryostat. Sections were analyzed for visual detection of cannula tracks and ink spread. Animals were excluded from analysis if ink appeared in the fourth ventricle or was misplaced in a neighboring parenchymal structure. All procedures were conducted as approved by the Institutional Care and Use Committee of University of Pennsylvania.

### Food and kaolin intake experiments

Rats were habituated to single housing in their home cage and received habituation (vehicle) injections at least 1 week before experimentation. Unless stated otherwise, food was removed from cages for 2 hours before dark cycle onset to reduce the influence of recent meals on food intake. Ex-4 (Bachem) and Ex-9 (Bachem) were dissolved in artificial cerebral spinal fluid, CNQX (Tocris)/MK-801(Tocris) was dissolved in 50% dimethyl sulfoxide (Sigma-Aldrich), and semaglutide (Cayman Chemical Company) was dissolved in its vehicle [40 mM tris HCl buffer and 0.01% Tween 20 (pH 8.0)]. Doses were chosen on the basis of previous studies to be subthreshold for ventricular effects (0.025 and 0.05 μg of Ex-4) or suprathreshold (10 μg of Ex-9, 10 nmol of semaglutide, and 0.05 μg CNQX/0.3 μg MK-801) for effect on feeding behavior (*26*). LiCl (0.15 M in 0.9% saline and 20 ml/kg; Sigma-Aldrich) was also injected intraperitoneally at a dose reported to cause visceral malaise and food intake suppression (*46*). All central injections were administered via bilateral LC-direct microinfusions at a volume of 100 nl per hemisphere. All peripheral injections were delivered intraperitoneally. All injections were made at dark cycle onset in a within-subjects design. For experiments with two drug injections, pretreatments were made 30 min (CNQX/MK-801 or vehicle) or 60 min (Ex-9 or vehicle) before the second injection.

Cumulative chow and kaolin readings were made at 1, 3, 6, and 24 hours after injection. Changes in body weight were calculated at the 24-hour time point. For meal pattern analysis, rats living in a BioDAQ system (Research Diets Inc) were injected similarly. The BioDAQ system records on a second-by-second basis for undisturbed measurements of episodic food intake. Individual bouts are initiated by the animal at onset and termination of feeding; bouts are separated by a 5-s interbout interval. A meal is defined as at least one bout with a minimum meal size of 0.2 g and separated by a 15-min undisturbed intermeal interval. Cumulative food intake, average meal size (grams per meal), and average meal frequency were calculated for the light cycle (12 hours) and dark cycle (12 hours). All behavioral experiments used a within-subjects, Latin-square design, with each treatment separated by 72 hours.

### Telemetry experiments

Telemetric transponders (HRC 4000 VitalView; STARR Life Sciences) for continuous monitoring of HR, core body temperature, and physical activity were inserted into the abdominal cavity of rats. Following postoperative recovery, food was removed for rats for 2 hours before the dark cycle. Rats were injected with either vehicle or Ex-4 (0.05 μg/100 nl) at dark cycle onset and placed back in their home cage. Data were collected for 24 hours and combined into 3-hour bins for analysis using VitalView Telemetry software (STARR Life Sciences). All rats received both drug treatments separated by 72 hours.

### Pupillometry

Rats were food deprived for 2 hours before experimentation, anesthetized using isofluorane (Butler Schein Animal Health), and placed in a stereotaxic frame with a camera (GoPro) aimed at their right eye. Five minutes of baseline data was collected followed by intraperitoneal injections of clonidine (0.1 mg/kg, Sigma-Aldrich) or vehicle. Recordings continued for 10 min following drug treatment. Rats received both treatments separated by 72 hours.

A separate group of rats (*n* = 7) was prepared with a guide cannula targeting the LC. Following postoperative recovery, rats were tested similarly. Here, injections consisted of 0.05 μg of Ex-4 or vehicle and recordings continued for 30 min following drug injection. Rats received both treatments separated by 72 hours. Pupillometry data were analyzed using ImageJ to measure pupil and iris diameters during still images captured from videos at specified time points. Baseline measurements represent the average pupil-to-iris ratio calculated from five still images taken 1 min apart during the 5 min before drug treatment. All other measurements were taken from a single still image taken at the specified time point following drug treatment.

### ELISA measurements of corticosterone and acetaminophen

To measure the effect of LC GLP-1R activation on stress hormone responses, we used an ELISA for plasma corticosterone. Food was removed from rats 2 hours before dark cycle onset. At dark cycle onset, rats received microinjections of either vehicle or Ex-4 (0.05 μg/100 nl) in the LC, and tail blood (100 μl) was collected 90 min after administration. Blood was collected into precoated EDTA microvette tubes (Sarstedt, supplier Thermo Fisher Scientific) on ice and subsequently centrifuged at 4° for 7 min at 5000 rpm. Plasma was collected and stored at 80°C until further processing. Plasma corticosterone concentration was analyzed in duplicate using a Corticosterone ELISA Kit (Enzo) according to the manufacturer’s provided instructions. Briefly, plasma was diluted 1:40 using an enzyme immunoassay buffer. Samples and standards (100 μl) were added to a well plate and incubated in antibody for 2 hours at room temperature. The plate was washed and conjugate, substrate solution and stop solution were added to the wells to terminate the reaction. The plate was read immediately using a plate reader (Tecan Sunrise) according to the manufacturer’s instructions. Assay sensitivity was 27 pg/ml. Corticosterone levels are expressed as nanograms per milliliter of plasma. All rats received both treatments separated by 72 hours.

To measure the effect of LC GLP-1R activation on gastric emptying, we used an ELISA for plasma acetaminophen. Rats were habituated to gavage for 5 days preceding the experiment. On experimental days, food was removed 5 hours before dark cycle onset to limit the influence of recent meals on gastric emptying rates. One hour before dark cycle onset, rats received microinjections of either vehicle or Ex-4 (0.025 or 0.05 μg/100 nl) into the LC. At dark cycle onset, baseline tail vein blood (200 μl) was collected and rats were gavaged with 6 ml of a test meal of vanilla-flavored Ensure (Abbott Laboratories, 1.42 kcal/ml) containing 40 mg of acetaminophen (Sigma-Aldrich). Tail vein blood (200 μl) was collected at 30, 60, 90, and 120 min after dark onset/gavage with precoated EDTA microvette (Sarstedt, supplier Thermo Fisher Scientific) on ice. Tubes were centrifuged at 4° for 7 min at 5000 rpm, and plasma was collected and stored at 80°C until further processing. Plasma acetaminophen concentrations were analyzed in duplicate using a commercial kit (Cambridge Life Sciences) adapted for a multiwell plate reader (Tecan Sunrise), according to the manufacturer’s instructions. All rats received both treatments separated by 72 hours.

### Electrophysiological experiments

To visually identify LC NE neurons during patch clamp electrophysiology, we injected adult (12 weeks) male TH-Cre rats with a virus that expresses enhanced green fluorescent protein (EGFP) in the presence of Cre (AAV pCAG-FLEX-EGFP-WPRE; titer: 2 × 10^12^, Addgene). Approximately 8 to 12 weeks after injection, rats were anesthetized with isoflurane and brains were removed. The brainstem was placed in an ice-cold bath of artificial cerebral spinal fluid. Horizontal sections of the brainstem containing the LC were sectioned at 250 mm on a vibrating microtome (Leica VT1200S). Slices were cut with a sapphire knife (Delaware Diamond Knives, Wilmington, DE) and secured using a fine polyethylene mesh in a perfusion chamber with continuous perfusion of artificial cerebrospinal fluid (aCSF) bubbled with 95% O_2_ to 5% CO_2_ at 32°C.

We recorded in aCSF with the following ionic conditions (in millimolar): 125 NaCl, 3 KCl, 1.2 KH_2_PO_4_, 1.2 MgSO_4_, 25 NaHCO_3_, 2 CaCl_2_, and 10 dextrose, bubbled with 95% O_2_ to 5% CO2 and the internal (in millimolar): 6 NaCl, 4 NaOH, 130 K-gluconate, 11 EGTA, 1 CaCl_2_, 1 MgCl_2_, 10 Hepes, 2 Na_2_ATP, and 0.2 Na_2_GTP. On average, recorded NE neurons had access resistance of 14 ± 2 megohms and an average holding current of −71 ± 15 pA, consistent with reported resting membrane potentials between −50 and −60 mV (*120*).

Neurons were studied under voltage-clamp conditions with a MultiClamp 700A amplifier (Molecular Devices, Union City, CA) and held at *V*_H_ = −60 mV in whole-cell patch configuration. Only recordings with a series resistance of <20 megohms were used for experiments to ensure good access and maintenance of the voltage clamp. Signals were filtered with a 1 kHz Bessel filter and sampled at 20 kHz using Axon pClamp10 software (Molecular Devices). Voltage-clamp recordings of TH^+^ LC neurons were performed under conditions to isolate glutamate-mediated EPSCs. Our internal and external bath conditions produced an ECl− = *V*_H_ = −60 mV, thus minimizing the influence of GABAergic signaling. Analysis of EPSC waveforms was consistent with AMPA receptor–mediated glutamatergic events and confirmed in a subgroup of neurons with bath perfusion of the AMPA-type glutamate receptor antagonist NBQX (10 mM). Following a baseline period in standard aCSF, Ex-4 was bath applied for 5 to 10 min at a flow rate of 2 ml/min and recording chamber volume of approximately 1.5 ml.

### In situ hybridization experiments

We used FISH to examine *Glp1r*, *Grin1A*, and *GluA1* mRNA transcripts within DBH-expressing LC neurons. FISH provides high cellular resolution and is necessary here, as the GLP-1R antibody is notorious for its lack of specificity (*121*). Rats (*n* = 4) were anesthetized, and brains were removed and snap-frozen in hexane. Brains were coronally sectioned into three serial sets on a cryostat at 20 μm and mounted onto subbed slides before being placed in a vacuum chamber overnight. Tissue was processed according to the RNAscope Fluorescent Multiplex Assay kit protocol for fresh frozen tissue (Advanced Cell Diagnostics). Serial set 1 was processed with probes for *Glp1r* and *DBH*, serial set 2 was processed for *Grin1A* and *DBH*, and serial set 3 was processed for *GluA1* and *DBH*. All probes were specific to the rat (Advanced Cell Diagnostics). Slides were coverslipped using Fluoro-Gel with 4′,6-diamidino-2-phenylindole (DAPI; Electron Microscopy Sciences). Images were obtained on a fluorescent microscope (Keyence) using the protocol described above.

### Immunohistochemical experiments

To examine LC c-Fos evoked by peripherally delivered semaglutide, we compared c-Fos responses in rats treated with vehicle or semaglutide (10 nmol). Food was removed from rats 2 hours before injections and injections occurred at the onset of the dark cycle. Ninety minutes after injection, rats were anesthetized with ketamine (90 mg/kg)/xylazine (2.8 mg/kg)/acepromazine (0.72 mg/kg) cocktail and were transcardially perfused with 0.1 M phosphate-buffered saline (PBS) followed by 4% paraformaldehyde in PBS. Brains were extracted and stored in 4% paraformaldehyde for 24 hours before they were transferred to 20% sucrose for cryoprotection. Brains were sectioned on a cryostat in the coronal plane at 30 μm. Sections taken through the rostral-caudal extent of the LC were collected for processing. Briefly, sections were blocked in 0.1 M PBS containing 3% normal donkey serum and 0.3% Triton X-100 at room temperature. Sections were incubated in primary antibody [rabbit anti–c-Fos (1:1000; Cell Signaling Technology) + sheep anti-DBH (1:1000; Abcam)] overnight. Following a PBS rinse, sections were incubated in a secondary antibody solution containing donkey anti-rabbit Alexa Fluor 488 (1:500; Jackson Immunoresearch) and donkey anti-sheep Alexa Fluor 647(1:500; Jackson Immunoresearch) for 4 hours at 4°C. Sections were coverslipped with Fluoro-Gel containing DAPI (Electron Microscopy Sciences). Five slide-mounted coronal sections per animal spanning the rostral-caudal extent of the LC (−14.60 to –11.96 mm from bregma, according to the stereotaxic atlas of Paxinos and Watson) were selected by an experimenter blind to treatment for quantification. Sections were imaged at 20× using a Nikon 80i fluorescence microscope with NIS-Elements AR 3.0 software. An average count of DBH^+^ neurons that expressed c-Fos within the LC was quantified by two treatment-blind experimenters. Data are expressed as an average of the two independent counts.

### Quantification and statistical analyses

In the experiments conducted using a between-subject design, animals were randomly allocated to their relative treatment conditions. Treatment order was randomly selected for all within-subjects work. All biological tissues/samples were processed in a blind fashion. Behavioral data were analyzed using GraphPad Prism 8.0. Electrophysiological data were analyzed using Clampfit 10 (Molecular Devices) and MiniAnalysis (Synaptosoft). All data are expressed as means ± SEM. For all statistical tests, *P* < 0.05 was considered significant. The results of all statistical tests performed can be found in the supplementary Excel file. Body weight changes were calculated by subtracting the weight of the animal on the day of injection from the body weight at 24 hours after injection. Behavioral data were analyzed by repeated-measures one- or two-way analyses of variance (ANOVAs) followed by Newman-Keuls post hoc tests or unpaired two-tailed *t* test. Electrophysiology data were analyzed using paired two-tailed *t* tests or Wilcoxon signed-rank tests.

## Supplementary Materials

### This PDF file includes:

Legend for Supplementary Excel File

### Other Supplementary Material for this manuscript includes the following:

Supplementary Excel File
