# Supplementary material for: The locus coeruleus contributes to the anorectic, nausea, and autonomic physiological effects of glucagon-like peptide-1
Source: Sci Adv. 2023 Sep 20;9(38):eadh0980. doi: 10.1126/sciadv.adh0980 (PMC10511187; doi:10.1126/sciadv.adh0980)
Supplement: Supplementary file 1 — Legend for Supplementary Excel File [file sciadv.adh0980_sm.pdf]

Science Advances
AAAS

Supplementary Materials for

The locus coeruleus contributes to the anorectic, nausea, and autonomic physiological effects of glucagon-like peptide-1

Samantha M. Fortin *et al.*

Corresponding author: Samantha M. Fortin, safortin@pennmedicine.upenn.edu; Matthew R. Hayes, hayesmr@pennmedicine.upenn.edu

Sci. Adv. **9**, eadh0980 (2023)
DOI: 10.1126/sciadv.adh0980

This PDF file includes:

Legend for Supplementary Excel File

Other Supplementary Material for this manuscript includes the following:

Supplementary Excel File

Title for Supplementary Excel file: Statistical summaries for all experiments.
